# Characteristics and treatment strategies of aggressive angiomyxoma in women: A retrospective review of 87 cases

**DOI:** 10.3389/fsurg.2023.966971

**Published:** 2023-04-17

**Authors:** Yang Wang, Xiaoling Bu, Yanxia Liu, Yu Xing, Qing Tong

**Affiliations:** Gynecology Department, Dongfang Hospital, Beijing University of Chinese Medicine, Beijing, China

**Keywords:** aggressive angiomyxoma, clinical manifestations, female, gynecologic oncology, treatment strategy

## Abstract

**Objective:**

Aggressive angiomyxoma (AAM) is a rare kind of soft tissue tumor. The purpose of this study is to summarize the clinical manifestations and treatment strategy of AAM in women.

**Method:**

We searched the case reports on AAM in EMBASE, Web of Science and PubMed, China biomedical database, Wanfang database, VIP database, and China National Knowledge Internet from the start of database construction to November 2022 without any language restrictions in place. Then, the obtained case data were extracted, summarized, and analyzed.

**Result:**

A total of 74 articles were retrieved involving 87 cases. The age ranges of onset were 2–67 years. The median age at onset was 34 years. The size of the tumor varied greatly among individuals, and about 65.5% of them were asymptomatic. MRI, ultrasound, and needle biopsy were used for diagnosis. Surgery was the primary mode of treatment, but it was prone to relapse. Gonadotropin-releasing hormone agonist (GnRH-a) might be used to reduce the tumor size before the operation and prevent recurrence after the operation. For patients who are unwilling to receive surgical treatment, GnRH-a alone could be attempted.

**Conclusion:**

Doctors should consider the possibility of AAM in women with genital tumors. For surgery, it must be ensured that the negative surgical margin is recommended and achieved for preventing recurrence, but we should not ignore the impact of the excessive pursuit for a negative margin on the patient’s reproductive function protection and postoperative recovery. Long-term follow-up is necessary regardless of whether patients receive medical treatment or surgical treatment.

## 1. Introduction

Aggressive angiomyxoma (AAM) is a rare kind of soft tissue tumor that was first reported in 1983 (1). In the WHO classification of soft tissue tumors, published in 2003, it was classified as a benign tumor within tumors of uncertain differentiation and formally named deep (“aggressive”) angiomyxoma (2), and the 2020 version followed this classification. At present, the cognition of AAM is limited and the pathogenesis remains unclear, but it has the characteristics of invasive growth and local recurrence, which calls for attention. Although AAM is a rare disease, it affects women more than men. The proportion of male to female patients in AAM was reported to be approximately 1:6 (3). Usually, case reports and retrospective studies with small sample sizes are the main forms of publication. The lack of comprehensive knowledge of AAM in women prevents gynecologists from providing more appropriate therapeutic advice. This study comprehensively searched published case reports and systematically summarized the characteristics, diagnosis, treatment strategy, and prognosis of AAM in women.

## 2. Materials and methods

We searched case reports included in three English databases (EMBASE, Web of Science, and PubMed) and four Chinese databases (China biomedical database, CBM; Wanfang database; VIP database; and China National Knowledge Internet, CNKI) before November 2022, regardless of language restrictions. The search terms were “deep aggressive angiomyxoma” and “aggressive angiomyxoma” (Supplementary Material). All articles in complete records were included and analyzed, while articles in repeated publications were excluded. Then, we established a new database for the articles and cases included, including the parameters of gender, age, symptoms, location, tumor size, treatment, pathological characteristics, metastasis, prognosis, etc. One researcher independently extracted these data from the literature and recorded them in the database, and the other researcher checked the database against the source literature. If these two needed any clarifications, a third researcher would be consulted to resolve it together. Next, the data in the new database were analyzed to show the characteristics and treatment experience of AAM. In terms of statistics, the data were expressed as median (interquartile range) and mean ± standard deviation. The *T*-test was used for making a comparison of the mean between the two groups in line with the normal distribution pattern, and the non-parametric *M*–*U* test was used for determining non-compliance.

## 3. Results

A total of 74 articles were included (4–77), containing 87 cases. Among the 74 studies, 37 were in Chinese (4–6, 8–12, 14, 15, 17, 19, 21, 22, 25–31, 33–35, 39, 40, 43, 45, 46, 51–53, 55, 56, 60, 61, 77), 34 were in English (7, 13, 16, 18, 20, 23, 24, 32, 36, 38, 41, 42, 44, 47, 50, 54, 57–59, 62–76), and three were in Spanish (48, 49, 54). Most patients came from Asia (77.0%), especially China (60.1%), a few from Europe (13.8%) and North America (7.0%), and only very few were from Africa (1.1%) and South America (1.1%) (Supplementary Table).

### 3.1. Basic data of patients

#### 3.1.1. Age distribution

**Figure 1:**
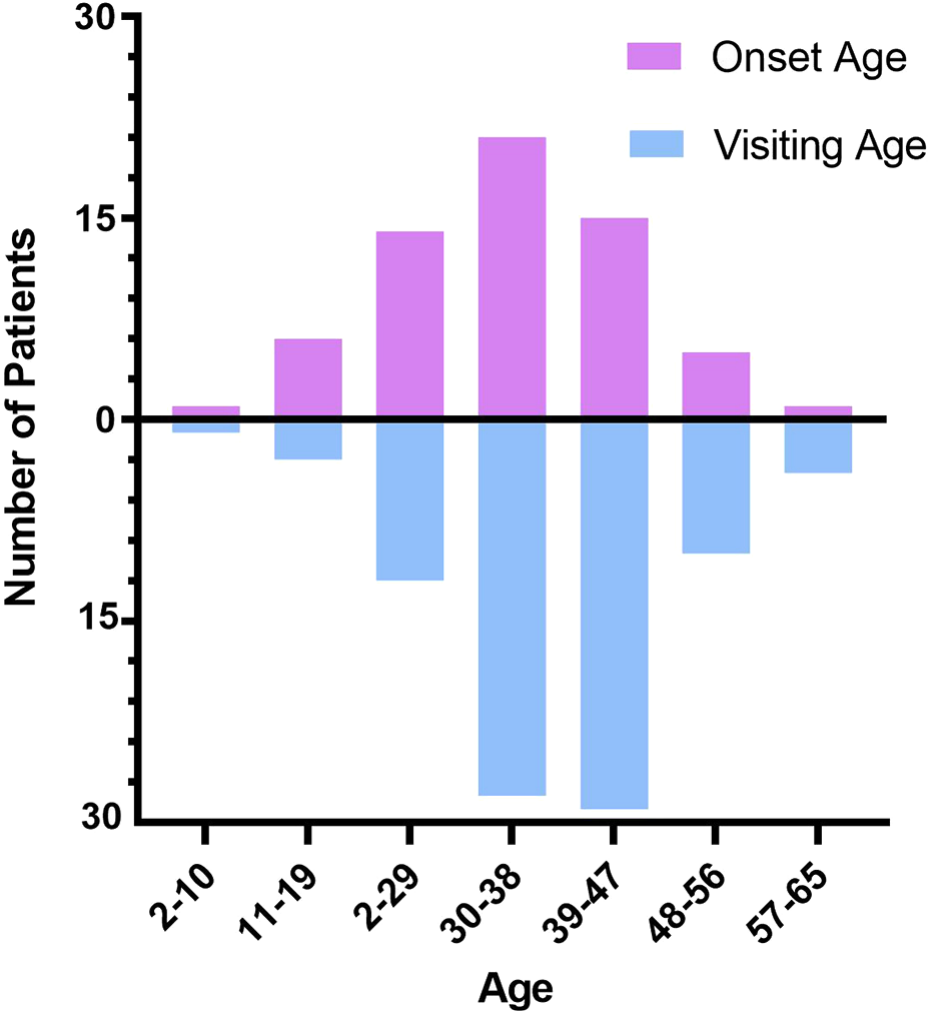
Age distribution of AAM.

The minimum visiting age of patients was two years and the maximum was 65 years. The median age was 37 (13) years, and the average age was 37.97 ± 10.45 [95% CI (35.74, 40.19)]. Then, we calculated the initial onset age (subtracting the number of years of the existence of the tumor from the age at first consultation according to the information obtained from the cases). A total of 63 patients were included. The minimum initial onset age was two years, the maximum onset age was 62 years, the median age was 34 (15) years, and the average age was (33.90 ± 1.35) [95% CI (31.20, 36.61)] (Figure 1).

### 3.2. Emergence or recurrence

Of the 87 patients, 72 were diagnosed with AAM for the first time, while the remaining 15 had a clear history of AAM resection, accounting for 19.7%. It is worth noting that among the recurrence patients, two were admitted after their fourth relapse (25, 49).

### 3.3. Clinical symptoms

**Figure 2:**
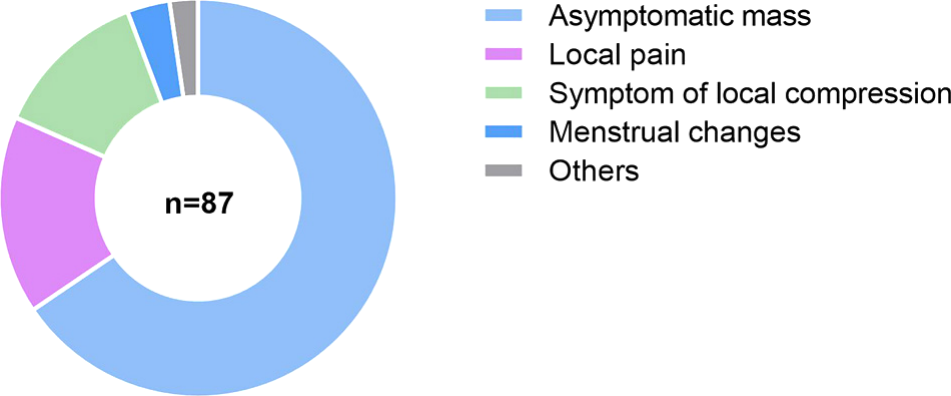
Clinical presentations of AAM. Symptoms of local compression: frequent micturition, dysuria, and difficult defecation; menstrual changes: cycle disorder and menorrhagia; others: partial body pain and breathing difficulty.

Among 87 patients, 57 (65.5%) were asymptomatic. Moreover, 30 patients (34.5%) suffered from different clinical presentations. Most of them had local pain, some of them showed symptoms related to local compression, and a few of them suffered abnormal menstruation (Figure 2). For asymptomatic patients, the interval between the first onset and treatment was (1.34 ± 0.35) years. Meanwhile, for those who showed clinical signs, the interval was (4.18 ± 0.67), which was significantly shorter than that of asymptomatic patients (*z* = −3.038, *P* = 0.002, <0.05).

### 3.4. Location

The most common location of AAM in the patients was the vulva, especially labium majus pudendi. In addition, among the lesions implicating the vulva, 39 were located in the unilateral vulva and two in the bilateral vulva. Then, we counted the left or right vulva. There were 22 patients (56.4%) who suffered left vulva AAM, which was more than the 17 (43.6%) who suffered right vulva AAM. It should be noted that in 10.3% of the reported cases, AAMs were found from more than one site, which, however, cannot be observed directly. In this situation, an imaging examination is necessary to determine the precise location of the tumor. The specific locations of AAM are shown in Table 1.

**Table 1:** Location of AAM.

Location	Case (*n*)	%
Pelvic cavity	14	16.1
Perineum	4	4.6
Vagina	9	10.3
Cervix	1	1.1
**Vulva**		
Mons pubis	1	1.1
Labium majus pudendi	26	29.9
Orificium vaginae	1	1.1
**Multiposition**		
Vulva extends into the pelvic cavity	9	10.3
Vulva extends into the perineum	5	5.7
Vagina extends into the pelvic cavity	5	5.7
Vagina extends into the perineum	2	2.3
Pelvic cavity and the lung	1	1.1
Pelvic cavity and the heart	1	1.1
Vulva and the lung	1	1.1
**Others**		
Scalp	1	1.1
Intracalvarium	1	1.1
Stomach	1	1.1
Low abdominal wall	1	1.1
Bladder	1	1.1
Right hip	2	2.3

### 3.5. Size and palpation characteristic

Among the tumors visible on the body surface, the tumor diameter ranged from 1 to 20 cm, with great variations in individual cases. However, most of the patients who came to the hospital had tumors of more than 4 cm in diameter. In addition, most tumors had no tenderness. The texture of AAM was medium or hard, usually with low activity. As to the question whether the AAM boundary was clear enough, different answers were reported from different cases.

### 3.6. Auxiliary examination

A total of 55 patients underwent imaging examination before treatment, including ultrasound, computed tomography (CT), and magnetic resonance imaging (MRI). Some patients underwent more than one type of imaging examination.

The results of ultrasonography were reported in 29 cases. It is worth noting that one case was diagnosed as AAM preliminarily by ultrasonography (53). A total of 24 patients underwent CT examination, and no one was diagnosed with AAM. MRI findings were reported in 28 cases. By MRI, AAM was considered the initial diagnosis in three patients (51, 60, 61). The features of the imaging examination are shown in Table 2.

**Table 2:** The features of the imaging examination of AAM.

Imaging examination	
Ultrasonography	Majority: cystic hypoechoic or isoechoic Minority: uneven medium and low mixed echoes
	With probe pressure: flow sensation
	CDFI: blood flow signals can be detected around the tumor
CT	Low-density soft tissue shadow After contrast administration: slightly irregular and enhanced
MRI	T1WI: hypointensity, lower than or equal to the muscle tissue T2WI: hyperintense, hypointensity bands arranged in layers and vortices, forming “laminated” or ‘swirled” appearance
	After contrast administration: gradual progressive enhancement

CT, computed tomography; MRI, magnetic resonance imaging.

Five patients received needle biopsy, and AAM was pathologically diagnosed in two (61, 69). Furthermore, one patient was pathologically diagnosed with spindle cell tumor and immunohistochemistry was required to be conducted on this patient to confirm the diagnosis (53). Meanwhile, the pathologic result of needle biopsy was uncertain in one patient (40).

### 3.7. Primary diagnosis

Among the first-onset cases (65 cases), only seven patients were initially diagnosed with AAM, for which different auxiliary examinations were required. Three patients underwent needle biopsy (61, 68, 69), four MRI (51, 60, 61, 68), and one ultrasound (53). For patients with incorrect initial diagnosis, bartholin's cyst or abscess and lipoma were the common conditions considered in the preliminary diagnosis . The cases of misdiagnoses in the preliminary diagnosis are listed in Table 3.

**Table 3:** Misdiagnosis in preliminary diagnosis.

Misdiagnosis	Case (*n*)
Bartholin's cyst	6
Bartholin's abscess	1
Gartner cyst	1
Perineocele	3
**Other pathological types of tumors**	
Fibroma	1
Uterine fibroids	1
Lipoma	4

### 3.8. Pathological features

#### 3.8.1. Macroscopic view

Most patients received surgical treatment. Some of them showed the characteristics of the gross specimens observed by the naked eye. To summarize, the tumor was lobulated, spherical, or strip shaped. The surface was smooth with a grayish red. Most of them had no obvious capsule or had an incomplete capsule. The section was grayish red, grayish white, or yellowish white, gelatinous, and of uniform texture.

#### 3.8.2. Microscopic view

We summarized the characteristics of the pathological sections under a light microscope. The tumor cells were spindle shaped, star shaped, or irregular, and scattered and loosely distributed in the myxoid matrix. The cells usually had no obvious atypia and division image, and many thin-walled or thick-walled blood vessels of different sizes could be observed.

#### 3.8.3. Immunohistochemistry result

Immunohistochemical results were obtained in 42 cases, and we classified all weak positives as positive. Among the indices detected by immunohistochemistry, vimentin had the highest expression rate, which reached 100%. It is worth noting that in the three patients with lung metastasis and heart metastasis, estrogen receptor (ER) and progesterone receptor (PR) of the tumor were strongly positive (16, 37, 50), and one of the patients eventually died (16). In another case of death, the tumor was located in the pelvic cavity and its ER and PR exhibited a strong positive expression (7). In addition, Ki-67 antigen was detected in 12 patients, and the Ki-67 index was reported in 10, amounting to less than 5% of cases, of which six cases constituted less than 1%. The immunohistochemical main antigen expression of AAM is exhibited in Table 4.

**Table 4:** Immunohistochemical main antigen expression of AAM.

Antigen	Number of positive cases	Number of reported cases	Positive rate (%)
CD 34	20	23	87.0
Desmin	25	29	86.2
ER	37	38	97.4
PR	34	37	91.9
Smooth-muscle actin	21	31	67.7
S-100	5	32	15.6
Vimentin	23	23	100.0
**Others**			
Actin	3	6	50.0
CD 117	0	3	0
CD 31	0	1	0
CK	0	5	0
EMA	0	1	0
H-caldesmon	1	2	50.0
β-Catenin	1	1	100.0

CD, cluster of differentiation; ER, estrogen receptor; PR, progesterone receptor; CK, cytokeratin; EMA, epithelial membrane antigen.

### 3.9. Treatment

In the included cases, surgery was the main treatment for AAM. The main operation method was local tumor resection. The surgical approach was related to the scope of tumor involvement. Two patients underwent radical vulvar surgery after local vulvar tumor resection, because the pathological diagnosis was AAM (21, 32). In two other patients, doctors suggested that patients receive supplementary and tissue expansion surgery, but the patients refused. However, one patient had a recurrence after 1 year and 6 months (31). Among the patients who were hospitalized for recurrence, two suffered massive bleeding during the first operation, and therefore, the tumors were not removed completely (5, 33). Meanwhile, positive margins were reported in three patients, for which patients did not receive an extended surgery. Among them, one patient did not relapse after 4 months of follow-up (42), one relapsed after 6 months (44), and one did not report the follow-up results (39). Two patients had multiple recurrences of vulvar AAM (recurrence three times (27) and four times (25), respectively), and both underwent bilateral inguinal and pelvic lymph node dissection, but unfortunately, it was not known whether they relapsed again after the surgery.

Although surgical treatment was commonly used, one AAM patient was reported to inject Leuprorelin subcutaneously without surgery, a type of gonadotropin-releasing hormone agonist (GnRH-a), at a dosage rate of 3.75 mg per month for 4 consecutive months after being diagnosed with pelvic AAM by puncture pathology. Remarkably, the symptoms caused by tumor compression in the vagina and anus almost disappeared (18). In addition, there were two patients who had relapsed after surgery. They refused to operate again, and the tumor was controlled by utilizing GnRH-a (13, 20).

Non-surgical treatment combined with surgery for AAM is increasingly used in clinical practice. Preoperative drug treatment is aimed to reduce the focus area. One patient with a 2 cm tumor in the vulva was treated with GnRH-a (Zoladex) at 3.6 mg per month for 3 months. The tumor disappeared partially, and then she received surgery (32). Among the included cases, the number of postoperative treatment cases was much higher than those before the operation. The purpose of postoperative therapy is to prevent recurrence. Postoperative therapy includes radiotherapy and drug therapy. Only one study published in 1997 reported a recurrent vulvar AAM patient who underwent postoperative local radiotherapy (5), and she did not relapse during the 8-year follow-up. The remaining patients were treated with drugs after surgery. The most common drug was GnRH-a, and the treatment cycle ranged from 3 months to 2 years (20, 24, 62, 74, 76). Most patients were started on GnRH-a immediately after the operation. However, one was given GnRH-a at 6 months postoperatively (23). It is worth noting that one patient had no recurrence during the treatment with GnRH-a for 12 months, but the tumor recurred 3 months after treatment withdrawal. Subsequently, the patient received another round of treatment with GnRH-a, and the recurrent tumor disappeared completely again (20). In addition, Letrozole, Tamoxifen, and Raloxifene were used to prevent recurrence (Table 5).

**Table 5:** Postoperative drug therapy.

Author, year	Age	ER, PR	Name of drug	Dose	Course of treatment	Follow-up result
Shinohara 2004 (20)	34	ER (+), PR (+)	GnRH-a	NA	12 months	Recurrence, 3 months after drug withdrawal
Alobaid 2005 (24)	30	ER (+), PR (+)	GnRH-a	Once every other month	6 months	No recurrence in 9 months
Hidalgo-Zambrano 2020 (59)	46	ER (+), PR: NA	GnRH-a	NA	NA	NA
Xu 2020 (62)	25	ER (+), PR (+)	GnRH-a	3.75 mg/month	3 months	No recurrence in 9 months
Akhavan 2021 (63)	33	NA	GnRH-a	NA	NA	No recurrence in 6 months
Elsaqa 2022 (72)	40	ER (+), PR: NA	GnRH-a	NA	NA	No recurrence in 6 months
Goyal 2022 (74)	40	ER (+), PR (+)	GnRH-a	3.75 mg/month	3 months	No recurrence in 1 year
Narang 2022 (76)	18	ER (+), PR (+)	GnRH-a	12.5 mg/3 month	2 years	No recurrence in 1 year
Husso 2017 (50)	61					No recurrence in 18 months
Peterknecht 2021 (67)	65	ER (+), PR (+)	Letrozole	NA	NA	NA
Herrera-Castro 2017 (49)	39	ER (+), PR (+)	Tamoxifen	NA	NA	NA
Srivastava 2021 (68)	37	ER (+), PR (+)	Tamoxifen	20 mg/day	NA	No recurrence in 2 years
Abu 2005 (23)	46	ER (+), PR (+)	Raloxifene	NA	NA	NA
Palomba 2011 (36)	32	ER (+), PR (+)	GnRH-a + Raloxifene	GnRH-a: 3.75 mg/28 days, Raloxifene: 180 mg/day	NA	No recurrence in 2 years
Zamani 2021 (70)	28	NA	Decapeptide	NA	NA	NA

NA, not available.

### 3.10. Prognosis and recurrence

Although AAM is prone to relapse, the prognosis of most patients is generally good. Only two cases reported that the patients eventually died (7, 16). The main cause of death was long-distance metastasis. The lesions initially located in the pelvis were then accompanied by multiple metastases. The metastatic sites were mainly distributed in the thoracic cavity and abdominal cavity, including the peritoneum, mediastinum, inferior vena cava, right atrium, and both lungs.

Follow-up information was recorded in 40 patients after the operation. Although 32 patients did not have recurrence during the follow-up period, the follow-up time varied greatly from 1 month to 6 years. In contrast, eight patients had recurrence during the follow-up period and none of them received any treatment after the surgery. Their ages ranged from as early as 2 to 48, which mainly included those of reproductive age. The shortest recurrence time was 1 month and the longest was 4 years after the operation. Examining the patients’ medical history in the case record, we found that the shortest recurrence time was 1 month and the longest recurrence time was 20 years. These patients were predominantly of reproductive age, but also included a 57-year-old postmenopausal woman.

## 4. Discussion

Aggressive angiomyxoma is a rare kind of soft tissue tumor. The etiology of AAM remains unknown. One early study found that 40% of metaphase cells of AAM patients suffered X chromosome loss (45, X), which may be related to the pathogenesis (78). Because of the higher incidence rate in women, our study focuses on female AAM patients. These patients come from all over the world, but mainly from Asian countries, especially China and India. This may be related to the large population size of China and India.

Recent studies suggest that AAM is a female hormone–dependent tumor (79, 80). However, some cases do not support this viewpoint. In this study, we found that the youngest AAM patient was only 2 years old, and another girl was 13 years old. In addition, a 67-year-old postmenopausal woman suffered from AAM. Our study showed that the median age of AAM women was 34 years, indicating that AAM mainly occurred in reproductive age. However, the distribution of visiting age suggested that delayed medical treatment was a common phenomenon, which may be related to the fact that most such patients feel no discomfort. In the early stages, most patients may have no symptoms, but the tumor size gradually increases at every stage and patients might suffer from discomfort caused by a local oppression of the tumor, such as frequent urination, urgent urination, walking discomfort, and so on. Our results indicated that AAM in women often invaded the vulva or pelvic cavity. The tumor of the vulva may be connected to the pelvic cavity, which cannot be found directly through observation or palpation, which may make us ignore the severity of the condition. Therefore, it is necessary to carry out an imaging examination. Otherwise, it can be easily misdiagnosed or would lead to unnecessary multiple operations. Misdiagnosis mainly occurs in AAM manifesting as a vulvar mass, including bartholin's cyst or abscess, local lipoma, and perineal hernia. Therefore, clinicians should factor-in AAM when confronted with atypical vulvar masses.

For the imaging examination, we discovered that experienced radiologists could diagnose AAM through ultrasound or MRI, whereas CT may not be beneficial in the diagnosis of AAM. However, irrespective of the type of imaging examination performed, it is helpful to gauge the scope of the tumor and consider the possibility of distant metastasis, which becomes an important component of the treatment strategy. The pathological examination of AAM is the gold standard for diagnosis. Ultrasound-guided needle biopsy can help extract a small amount of tissue to make pathological sections, which could aid in the diagnosis.

At present, surgery remains the main treatment option for AAM. The surgical approach is related to the range of tumor involvement. As the study is limited by size, our results cannot help us draw a conclusion about whether a positive tumor surgical margin would impact the recurrence rate. However, a retrospective study with the largest number of cases (106 cases) pointed out that there was no significant difference in the recurrence-free rate between patients with negative and positive margins within 10 years after operation (3). Therefore, we suggest that when the tumor involves a wide range, it is not necessary to blindly pursue the strategy of achieving a negative margin or expand the scope of secondary surgery; otherwise it will lead to surgical trauma and affect the preservation of fertility and quality of life of postoperative patients. In addition, AAM of the pelvic cavity and vulva was found in two patients during pregnancy (36, 73). Therefore, it is not recommended to remove the tumor during the cesarean section. Such removal would entail the risk of massive bleeding. Although surgery remains the main way to treat AAM, for patients who are unwilling to receive surgery, GnRH-a could be a new treatment therapy to alleviate symptoms and reduce tumor size.

In order to reduce the recurrence rate, postoperative medication and regular follow-up are highly important. For patients of reproductive age with a positive ER of tumor, the postoperative application of estrogen receptor modulators (Tamoxifen and Raloxifene), aromatase inhibitor (Letrozole), and GnRH-a would help reduce the recurrence rate. However, the course of drug use needs further study.

There are some limitations to our study. Some articles were excluded because of insufficient clinical data. Also, we excluded cross-sectional studies because they did not contain direct and detailed case information. These factors limited the number of articles included. Thus, we are unable to provide definite answers to some questions such as whether a positive tumor surgical margin would impact the recurrence rate. Moreover, because of the fact that AAM is not a common type of tumor, there exists a possibility that some cases cannot be diagnosed and reported, which may lead to publication bias.

In conclusion, when women have atypical vulvar tumors, a diagnosis of AAM should be considered. MRI and ultrasonography or pathological needle biopsy are helpful to determine the tumor scope and diagnose AAM. Surgery remains the main treatment choice for AAM. The tumor focus area should be reduced as much as possible during operation. However, in the case of a wide range of tumor invasion, it is futile to blindly pursue the negative margin strategy and cause unnecessary intraoperative damage, which is not conducive to the postoperative repair and fertility preservation of patients. GnRH-a could be recommended for AAM patients who refuse surgery or for those in whom there is a need for reducing the tumor size before operation. Patients with local recurrence can still consider GnRH-a treatment to avoid secondary surgery. Long-term and regular follow-up by performing an imaging examination is necessary.

## Author contributions

YW was involved in protocol development, data collection, and manuscript writing. XB was involved in data collection and performed data analysis. YL was responsible for data management and data analysis. YX carried out manuscript editing. QT was involved in protocol development and did the manuscript editing. All authors contributed to the article and approved the submitted version.
