# Supplementary material for: Characteristics and treatment strategies of aggressive angiomyxoma in women: A retrospective review of 87 cases
Source: Front Surg. 2023 Apr 17;10:966971. doi: 10.3389/fsurg.2023.966971 (PMC10149767; doi:10.3389/fsurg.2023.966971)
Supplement: Supplementary file 1 [file Table1.docx]

Supplementary Table: The Basic Characteristic of Studies

| Author year | Country | Language | Number of Included Case |
| --- | --- | --- | --- |
| Dai 1994 (4) | China | Chinese | 1 |
| Lu 1997 (5) | China | Chinese | 1 |
| Tan 1998 (6) | China | Chinese | 2 |
| Siassi 1999 (7) | German | English | 1 |
| Li 2000 (8) | China | Chinese | 1 |
| Ma 2000 (9) | China | Chinese | 1 |
| Mao 2000 (10) | China | Chinese | 1 |
| Zhang 2000 (11) | China | Chinese | 1 |
| Zhao 2000 (12) | China | Chinese | 3 |
| Fine 2001 (13) | US | English | 1 |
| Wang 2001 (14) | China | Chinese | 1 |
| Zhang 2001 (15) | China | Chinese | 3 |
| Blandamura 2003 (16) | Italy | English | 1 |
| Huang 2003 (17) | China | Chinese | 1 |
| Poirier 2003 (18) | Canada | English | 1 |
| Wang 2003 (19) | China | Chinese | 1 |
| Shinohara 2004 (20) | Japan | English | 1 |
| Su 2004(21) | China | Chinese | 1 |
| Yu 2004 (22) | China | Chinese | 1 |
| Abu 2005 (23) | UK | English | 1 |
| Alobaid 2005 (24) | Canada | English | 3 |
| Shi 2005 (25) | China | Chinese | 1 |
| Luo 2006 (26) | China | Chinese | 3 |
| Ren 2006 (27) | China | Chinese | 3 |
| Ling 2007 (28) | China | Chinese | 1 |
| Liu 2007 (29) | China | Chinese | 1 |
| Yang 2007 (30) | China | Chinese | 1 |
| Li 2008 (31) | China | Chinese | 2 |
| Sereda 2009 (32) | Canada | English | 1 |
| Jiang 2010 (33) | China | Chinese | 1 |
| Feng 2011 (34) | China | Chinese | 1 |
| Lang 2011 (35) | China | Chinese | 1 |
| Palomba 2011 (36) | Italy | English | 1 |
| Geng 2012 (37) | China | English | 1 |
| Sirasagi 2012 (38) | India | English | 1 |
| Chen 2013 (39) | China | Chinese | 1 |
| Gao 2013 (40) | China | Chinese | 1 |
| Huang 2013 (41) | China | English | 1 |
| Kiran 2013 (42) | Turkey | English | 1 |
| Liu 2013 (43) | China | Chinese | 1 |
| Mallick 2013 (44) | India | English | 1 |
| Qian 2013 (45) | China | Chinese | 1 |
| Wang 2013 (46) | China | Chinese | 1 |
| Abu 2015 (47) | Ireland | English | 1 |
| Guldris Nieto 2015 (48) | Spain | Spanish | 1 |
| Herrera-Castro 2017 (49) | Spain | Spanish | 1 |
| Husso 2017 (50) | Finland | English | 1 |
| Chao 2018 (51) | China | Chinese | 1 |
| Shang 2018 (52) | China | Chinese | 1 |
| Wu 2018 (53) | China | Chinese | 1 |
| Carlos Manterola 2019 (54) | Spain | Spanish | 1 |
| Guo 2019 (55) | China | Chinese | 1 |
| Zhang 2019 (56) | China | Chinese | 1 |
| Alomary 2020 (57) | Saudi Arabia | English | 1 |
| Amante 2020 (58) | Portugal | English | 1 |
| Hidalgo-Zambrano 2020 (59) | Colombia | English | 1 |
| Ma 2020 (60) | China | Chinese | 1 |
| Wang 2020 (61) | China | Chinese | 2 |
| Xu 2020 (62) | China | English | 1 |
| Akhavan 2021 (63) | Iran | English | 1 |
| Altinmakas 2021 (64) | Turkey | English | 1 |
| Djusad 2021 (65) | Indonesia | English | 1 |
| Patrick 2021 (66) | India | English | 1 |
| Peterknecht 2021 (67) | UK | English | 1 |
| Srivastava 2021 (68) | India | English | 1 |
| Yang 2021 (69) | China | English | 1 |
| Zamani 2021 (70) | Iran | English | 1 |
| Cao 2022 (71) | China | English | 1 |
| Elsaqa 2022 (72) | Egypt | English | 1 |
| Espejo-Reina 2022 (73) | Spain | English | 1 |
| Goyal 2022 (74) | India | English | 1 |
| Muskan 2022 (75) | Nepal | English | 1 |
| Narang 2022 (76) | India | English | 1 |
| Tang 2022 (77) | China | Chinese | 1 |
