# Supplementary material for: Characteristics and treatment strategies of aggressive angiomyxoma in women: A retrospective review of 87 cases
Source: Front Surg. 2023 Apr 17;10:966971. doi: 10.3389/fsurg.2023.966971 (PMC10149767; doi:10.3389/fsurg.2023.966971)
Supplement: Supplementary file 3 [file Table3.docx]

**APPENDICES**

**Search strategies**

**Embase**

#1 deep aggressive angiomyxoma:ab,ti

#2 aggressive angiomyxoma:ab,ti

#3 OR/1-2

**Web of Science**

#1 deep aggressive angiomyxoma: topic

#2 aggressive angiomyxoma: topic

#3 OR/1-2

**Pubmed**

#1 deep aggressive angiomyxoma: ti,ab

#2 aggressive angiomyxoma: ti,ab

#3 OR/1-2

**China biomedical database (CBM)**

#1 深部侵袭性血管粘液瘤: 常用字段

#2 侵袭性血管粘液瘤: 常用字段

#3 OR/1-2

**Wanfang database**

#1 深部侵袭性血管粘液瘤: 主题

#2 侵袭性血管粘液瘤: 主题

#3 OR/1-2

**VIP database**

#1 深部侵袭性血管粘液瘤: 题名或关键词

#2 侵袭性血管粘液瘤: 题名或关键词

#3 OR/1-2

**China National Knowledge Internet (CNKI)**

#1 深部侵袭性血管粘液瘤: 主题

#2 侵袭性血管粘液瘤: 主题

#3 OR/1-2
